# Evolution of a longitudinal, multidisciplinary, and scalable patient navigation matrix model

**DOI:** 10.1002/cam4.2950

**Published:** 2020-03-04

**Authors:** Cheyenne M. Corbett, Tamara J. Somers, Christine M. Nuñez, Catherine M. Majestic, Rebecca A. Shelby, Valarie C. Worthy, Nadine J. Barrett, Steven R. Patierno

**Affiliations:** ^1^ Duke Cancer Institute Duke University School of Medicine Durham NC USA; ^2^ Department of Psychiatry and Behavioral Sciences Duke University School of Medicine Durham NC USA; ^3^ Department of Family Medicine and Community Health Duke University School of Medicine Durham NC USA; ^4^ Department of Medicine Duke University School of Medicine Durham NC USA; ^5^ Miller School of Medicine University of Miami Miami FL USA

**Keywords:** patient navigation

## Abstract

This Longitudinal patient navigation Matrix Model was developed to overcome barriers across the cancer care continuum by offering prepatients, patients, and their families with support services. The extraordinary heterogeneity of patient needs during cancer screening, risk assessment, treatment, and survivorship as well as the vast heterogeneity of oncology care settings make it nearly impossible to follow a static navigation model. Our model of patient cancer navigation is unique as it enhances the traditional model by being highly adaptable based on both patient and family needs and scalable based on institutional needs and resources (eg, clinical volumes, financial resources, and community‐based resources). This relatively new operational model for system‐wide and systematic navigation incorporates a carefully cultivated supportive care program that evolved over the last decade from a bottom up approach that identified patient and family needs and developed appropriate resources. A core component of this model includes shifting away from department‐centric operations. This model does not require a patient to opt in or independently be able to report their needs or ask for services—it is an opt out model. The multidisciplinary “cross‐training” model can also facilitate reimbursement and sustainability by clarifying the differentiating actions that define navigation services: identification of barriers to quality care and specific actions taken to overcome those barriers, across the full continue of cancer care from community engagement to survivorship or end‐of‐life care.

## Graphical abstract

Patient navigation aims to work at both the community level and the health system or clinic level. In this article, we describe the process of development and implementation of a model of a longitudinal navigation program that is a “to and through model” where navigators are responsible for identifying prepatient, patient, provider, community, and system barriers that compromise the seamless delivery of cancer care across the full cancer continuum and contribute to disparities in cancer incidence, morbidity, and mortality.

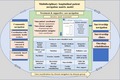

## 1 INTRODUCTION

Since its inception in Harlem, New York in 1990, patient navigation has established itself as a valuable element of cancer care and has undergone rapid national and international dissemination and implementation.^1^ Patient navigation was originally defined by the National Cancer Institute as “support and guidance offered to persons with abnormal cancer screening or a new cancer diagnosis in accessing the cancer care system, overcoming barriers, and facilitating timely, quality care provided in a culturally sensitive manner.”^2^ This relatively narrow definition emerged from the fact that, in its earliest forms, patient navigation was focused on the establishment of processes to help newly diagnosed cancer patients get faster and more efficient access to cancer care services, with a focus on targeting treatment barriers.^2^ Since then, the scope of patient navigation has expanded longitudinally to include community‐engaged navigation and survivorship navigation.^2^, ^3^ Patient navigation is often focused on patients who have the highest barrier burden and least access to care and is recognized and utilized to reduce cancer health disparities.^2^, ^3^, ^4^, ^5^, ^6^, ^7^

As patient navigation underwent rapid dissemination and uptake, a large number of different models evolved, each tailored to the individual needs of the health system, hospital, department, division, or disease group (ie, breast cancer) within which the navigation program was embedded. The size and type of the navigation program was, and in large part still is, determined by a constellation of a number of parameters, including availability and sustainability of resources, availability of local resources outside of the health system, type of organization, patient characteristics, geo‐demographic location of the facility and the patients, local barriers, programmatic needs, and the presence of an internal champion. These factors also largely determine the type and qualification level for the sought‐after patient navigator positions: nonclinical or lay navigator or clinical navigator (ie, nurse, social worker, or other clinician cross‐trained as a navigator). This lack of a cohesive model for patient navigation programs, and the fact that different aspects of navigation services can be delivered at many levels (lay to clinical) has led to ongoing confusion over how to define the role, scope of practice, and necessary training for a patient navigator.^8^

For this Commentary we embrace multilevel patient navigation and define the navigation aspect of any practitioner as that component of their job that focuses on identification of barriers to care (barrier assessment) and engagement in actions that help patients overcome those barriers. This transcends whether the navigation service is provided by nonclinical (lay) navigator in the community, by a licensed social worker, by a financial counselor, or by a nurse navigator. Navigators can serve a variety of roles in helping patients navigate the healthcare system to increase the likelihood of receiving culturally appropriate, high‐quality cancer care.^4^ For example, navigators may help patients with transportation problems, address psychosocial challenges related to fear or medical mistrust, resolve insurance issues, find appropriate healthcare providers from physicians to psychosocial therapists, accompany patients to appointments, explain treatment options in lay, culturally appropriate language, serve as a liaison with the healthcare team, help handle medical paperwork, and assist caregivers. Through eliminating barriers faced by patients, particularly those who are underserved, patient navigation programs aim to provide more timely access to screening, diagnosis, treatment, or supportive services, resulting in greater adherence to care and improved health outcomes.^2^, ^9^

In longitudinal models, patient navigation aims to work at both the community level and the health system or clinic level. There are numerous principles that patient navigation seeks to instill in communities and healthcare systems including making information and resources understandable, available, accessible, affordable, and appropriate.^10^ Research suggests that there are benefits to the implementation of patient navigation programs in cancer centers, including better patient‐provider communication and improved ability to identify patients with major barriers to receipt of care. Navigation may help patients better adhere to screening, leading to more timely diagnosis and overall cancer care.^2^, ^3^, ^7^, ^11^, ^12^, ^13^, ^14^, ^15^, ^16^ When patient navigation services are provided, patients also are more likely to be connected with appropriate treatment and supportive care services and receive referrals to establish treatment care plans.^17^ In addition, patient navigation has been shown to improve patient adherence to recommended cancer care resulting in less distress and fatigue, as well as improved the quality of life.^14^ Cancer patient navigation programs improve access to and usage of cancer services for underserved and minority patients.^9^, ^10^

There may also be positive outcomes for health systems that implement a patient navigation program. Through increased and timely screening, there may be an increase in life expectancy, as well as a decrease in costs due to earlier detection. For example, screening colonoscopies resulting from a navigation program have been shown to be cost effective.^18^ Increased care coordination through navigation can create cost savings by having less emergency department visits, reduced number of hospital admissions, less unneeded diagnostic testing, fewer no‐shows, and fewer patients lost to follow‐up.^17^, ^19^ By improving adherence to treatment plans, patients will be more effectively managed throughout the care continuum resulting in increased utilization of recommended services, improved follow‐up care, and more appropriately utilized hospice services.^4^, ^20^

There are many challenges in developing and sustaining optimal navigation programs, but one of the most difficult is the sheer complexity of identifying and addressing multilevel barriers to care, and the tendency to view a patient navigator as a catchall solution to an overly broad set of patient‐level and health system‐level problems. Patient navigators are often designated with responsibility for identifying practical, financial, and psychosocial barriers that present challenges to care. Practical barriers can include lack of transportation, housing, and childcare.^2^ Financial barriers include lack of insurance, financial concerns, and employment issues and are often most time consuming to resolve.^21^ Common attitudinal barriers are preconceived perceptions toward medical providers and cancer testing or treatment. Interpersonal barriers include a lack of social support, fear, low literacy, language concerns, and communication concerns with healthcare providers. Other medical and mental health comorbidities may play a role in patients’ motivation to seek services for cancer or their ability to participate in such services. These patient‐level factors are exacerbated by health system‐level factors including the fragmented nature of modality‐driven treatment models and the inefficiencies in care coordination that often accompany high patient volumes. Patient navigation could be a direct intervention for these barriers, but given the breadth of possible roles and responsibilities for patient navigators in most medical settings, it is difficult to optimally identify and fulfill the navigation needs of every patient. Limited resources and infrastructure, including funding sources, to address multiple patient needs is also an ongoing challenge for health systems considering patient navigation.

Patient navigation in routine clinical cancer care is lacking in many settings or presents challenges to fully operationalizing beyond subsets of patients, locations, or phases of cancer experience. Patient navigation models have typically worked well in smaller, lower volume settings, but in larger institutions and academic medical centers patient navigation programs are often unevenly distributed to, and molded by, pockets of the institution such as high‐risk breast cancer clinics. In further developing patient navigation as a recognized and reimbursable healthcare intervention, it will be important to establish practice guidelines, standardize training for patient navigators, and aim to support patients throughout the continuum of care from prediagnosis (eg, screening, health behaviors) to cancer survivorship (eg, rehabilitation, transition back to primary care), and end‐of‐life care.^4^, ^10^, ^17^

By targeting these various phases, patient navigators will be able to coordinate the appropriate interventions and resources for each phase.^17^ Nevertheless, in high‐volume cancer centers the major challenge, and optimum solution, lies in creating a seamless continuum of navigation across all phases. Patient navigation is also challenged by the distribution of limited resources to address patient needs across the cancer continuum. An optimal patient navigation system will have the resources and infrastructure to create an individual plan for overcoming the barriers to care, track patients, and continually reassess and modify the plan. This raises the challenge of how to fund patient navigation programs, and calls attention to the fact that until recently, the majority of active patient navigation programs are funded by grants that are time limited and therefore not sustainable, and relatively narrow in both scope and disease focus.

In this paper, we present a model that has been developed in a complex system managing the oncology service lines of (a) a high‐volume academic National Cancer Institute‐designated Comprehensive Cancer Center, (b) two entity‐owned and operated community hospitals, (c) an oncology joint venture with another nearby health system, and (d) a mid‐Atlantic regional network consisting of 14 additional community hospitals. Below we detail one approach to developing a patient navigation program in a high‐volume setting that is multidisciplinary, matrixed, and scalable in a way that institutions of any size can pick and choose components that best fit their resources and needs. A central goal was to create a sustainable program across the full cancer continuum, and a matrixed navigation culture wherein each provider‐patient touchpoint along the care pathway involves the core element of patient navigation; ongoing barrier and needs assessment and referral to the appropriate service to address those barriers and needs.

## 2 APPROACH

Prior to this effort, several cancer care loci within the system had evolved either without patient navigation or with different, limited models. We first created a transdisciplinary patient navigation Working Group to identify concerns, problems, and system‐wide challenges, and to develop, pilot test, and implement solutions. An extensive community needs assessment^22^ helped identify gaps in patient education and care utilization, especially in minority, uninsured, and underinsured patients. In response to this information, we executed a system‐wide gap analysis and needs assessment, examined existing available institutional resources, and conducted brainstorming sessions to envision the development of a seamless, scalable navigation program.^23^

**Figure 1:**
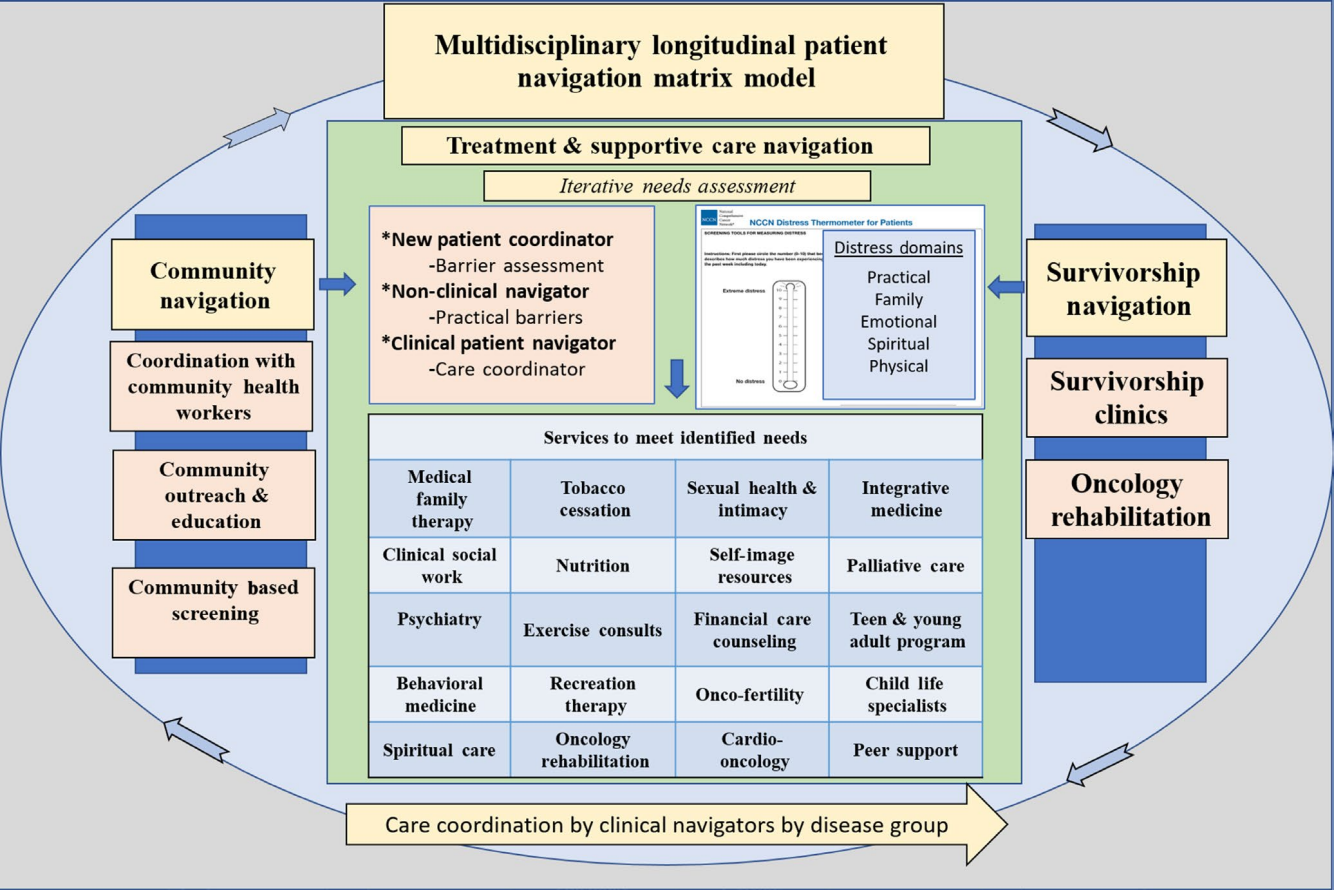
The multidisciplinary, longitudinal patient navigation matrix includes prepatient, patient, survivorship, and end‐of‐life care. Patients complete a distress screening at each oncology provider appointment and are referred to services as indicated

The culmination of these efforts was the creation and implementation of a longitudinal multidisciplinary patient navigation matrix. The matrixed program enables the identification and reduction of barriers at multiple patient‐provider touchpoints across the care continuum, and increased access to, and utilization of cancer screenings, treatment, and supportive care resources, in a model that is scalable to a high‐volume academic cancer center setting and adaptable to community‐based clinical settings. The developed patient navigation matrix model is longitudinal, extending throughout the cancer experience: from community‐engaged navigation providing education and connection to prevention and screening services; to care coordination during treatment; to navigation through survivorship and end‐of‐life care (Figure 1). The matrix is multidisciplinary in that it utilizes a number of different personnel, each cross‐trained in patient navigation and connected through the electronic medical record (EMR), positioned at key checkpoints, to assess barriers and assist either the prepatient or the patient in assessing their needs and connecting to services and resources.

## 3 LONGITUDINAL NAVIGATION CROSS‐TRAINING: WHO DOES WHAT, WHERE, AND WHEN?

### 3.1 Community‐engaged navigation

Improvements in cancer prognosis and decreased cancer‐related mortality have been shown to be influenced by several factors including access to care, as well as early detection and education; however, for many underserved communities (eg, lower socioeconomic status, racial minorities) services remain difficult to access.^24^, ^25^ The front end of our “to and through” navigation matrix model is staffed by community‐engaged navigators.^26^ One important task of community‐engaged navigators is to remove barriers to quality cancer care by educating individuals in the community about cancer with the goal of improving general cancer perception and knowledge and improving cancer screening rates.^9^, ^10^ Community‐engaged navigators also work with healthcare systems and providers to make them aware of barriers to cancer care in their center's catchment area and educate them about the community, and the social, cultural, and political factors that impact communication, adherence, and other aspects of patient care. Community‐engaged navigators help develop and maintain relationships with individuals, community organizations, and faith communities, and they are also able to facilitate screening referrals and follow‐up. On an annual basis our community‐engaged navigators, through various community events, reach over 3000 people with cancer education, resulting in 300‐400 touchpoints of barrier assessment and resolution, and 50‐100 people navigated to clinical follow‐up.

Community‐engaged navigators in our system are nonclinical and most often have at least a bachelor's level degree and/or additional training as community health workers. When on‐boarded they undergo training at one of the nationally recognized patient navigation training programs, and receive additional training specific to our system. They work in close coordination with city and county Departments of Health and Human Services and with State‐run programs employing Community Health Workers. Because these navigators are embedded within our health system, with access to our EMR, they can effectively navigate community “prepatients” to and through the access portals of the medical center. Community‐engaged navigators also interface with referring and community providers to ensure ease of access to oncology care and facilitate communication among providers and referring providers, thus navigating within and between systems.

### 3.2 Navigation from diagnosis to survivorship or end of life

The community‐engaged navigators also work in close association with a “navigation matrix” inside the cancer center, with the goal of creating a seamless mechanism for patient handoff. The treatment‐focused component of the patient navigation matrix is operationally embedded within and overseen by our institution's Supportive Care and Survivorship Center which serves as the nucleus for navigation once patients are receiving care at our site. Patient navigators play important roles during the treatment and survivorship phases of care, often addressing language barriers and connecting people with practical or financial resources (eg, housing, transportation). In our multidisciplinary matrix model, we cross‐train several nonclinical and clinical persons in patient navigation skills and practice, including new patient coordinators, financial counselors, clinical social workers, and medical family therapists.

Our “New Patient Coordinators” are the first line of patient navigation as a prepatient becomes a patient. These nonclinical navigators, whose principal role is to set appointments and prepare each patient for their first visit, receive in‐house training in the basic principles of patient navigation, particularly barrier assessment. In setting up the first appointment they are tasked to administer a scripted set of questions for high‐level barrier assessment, particularly in the domain of practical barriers, and immediate referral to a team of nonclinical navigators to address those needs. Our goal is to stratify patients by burden severity. For example, nonclinical navigators may address practical barriers such as arranging transportation, or refer new patients to navigation‐trained financial counselors to address practical solutions to cancer‐related financial challenges, or refer to navigation‐trained clinical social workers to assist patients with crisis‐oriented needs.

Patient navigators at our institution are required to hold a college degree appropriate to their domain of navigation. Navigators operating in the clinical arena are expected to have an accredited degree in an appropriate field (eg, nursing, clinical social work). With a multidisciplinary, multicultural, and bilingual staff, our patient navigators are able to address the diverse needs of the community and connect cancer patients with support services and resources. Uniquely, our Supportive Care and Survivorship Center's patient navigation matrix model includes a navigation‐trained psychosocial care team who all work together in a tiered model of support that provides the appropriate level and type of services to address the particular psychosocial needs or barriers. Our nonclinical navigators have direct access, from screening to survivorship, to refer patients to the number of services summarized in Table 1.

**Table 1:** This table provides a summary of services that navigators routinely refer patients to at each step of the Longitudinal Navigation Model

Services	
Medical family therapy	Medical family therapists work with individuals, couples, and families to address psychological, emotional, and social/relational needs related to cancer
Clinical social work	Clinical social workers assist patients and family members with emotional and practical concerns (eg, disability, medicare) related to cancer
Psychiatry	Psychiatrists conduct assessments and provide medication management and intensive therapy services for patients with mental health concerns related to cancer
Behavioral medicine	Clinical psychologists provide evidence‐based treatments for a range of clinical difficulties associated with cancer including: behavioral weight management, health behavior change, coping skills training for cancer‐related pain and fatigue, psychological distress, and brief cognitive screenings
Spiritual care	Chaplains and other spiritual professionals are available for spiritual assessment, care, and support, care planning, and spiritual health programs
Tobacco cessation	The tobacco cessation program helps cancer patients and their family members quit using tobacco products
Nutrition	Registered dietitians work with patients to help prevent malnutrition, improve digestive health, and minimize side effects related to cancer treatments
Exercise consults	Exercise physiologists provide consultations services and personalized exercise plans specialized for cancer patients.
Recreation Therapy	Recreational therapists offer services (eg, strength building, motivation, confidence) for patients and family members during in‐patient hospital stays
Oncology Rehabilitation	Exercise physiologist, lifestyle counselors, and lymphedema specialists are available to help patients during cancer treatment and during recovery
Sexual health and intimacy	Behavioral sexual health services include psychoeducation and psychotherapy focused on addressing concerns about sexual function, sexual feelings and intimacy, and changes in sexual health following treatment for cancer
Self‐image resources	Self‐image consultations and products are available through boutiques at the cancer centers
Financial care counseling	Financial care counselors work closely with patients to address billing and insurance questions, obtain preauthorizations before treatments, and inform patients about insurance coverage
Oncofertility	This services is provided by productive endocrinologists and fertility specialists, urologists, nurses, psychologists, and medical family therapists and works with cancer patients to understand their options, provide fertility services, and address associated physical, emotional, and financial concerns
Cardio‐oncology	This services provides cancer patients with services to assess their cardiology risk associated with their cancer and cancer treatments
Integrative medicine	Integrative medicine specialists to provide complementary services, such as acupuncture and massage therapy
Palliative care	The palliative care team works closely with patients and family members throughout all stages of illness and works to provide relief from pain, stress, and other symptoms related to their illness and to help obtain the best possible quality of life
Onco‐primary care	This service works to create a formal link between patients’ primary care physicians and the oncology team in the care of patients across the cancer continuum
Teen and young adult oncology	This program addresses the unique needs of teens and young adults with cancer: primarily working to meet the psychosocial needs of this age group
Child life services	Child life specialists help educate and support children (of adult oncology patients) and their families related to cancer diagnosis and treatment
Peer support	This service provides an opportunity for cancer patients to discuss cancer and receive support with someone like them who has had a similar cancer experience

### 3.3 Distress screening triage: An iterative assessment of patient needs

**Figure 2:**
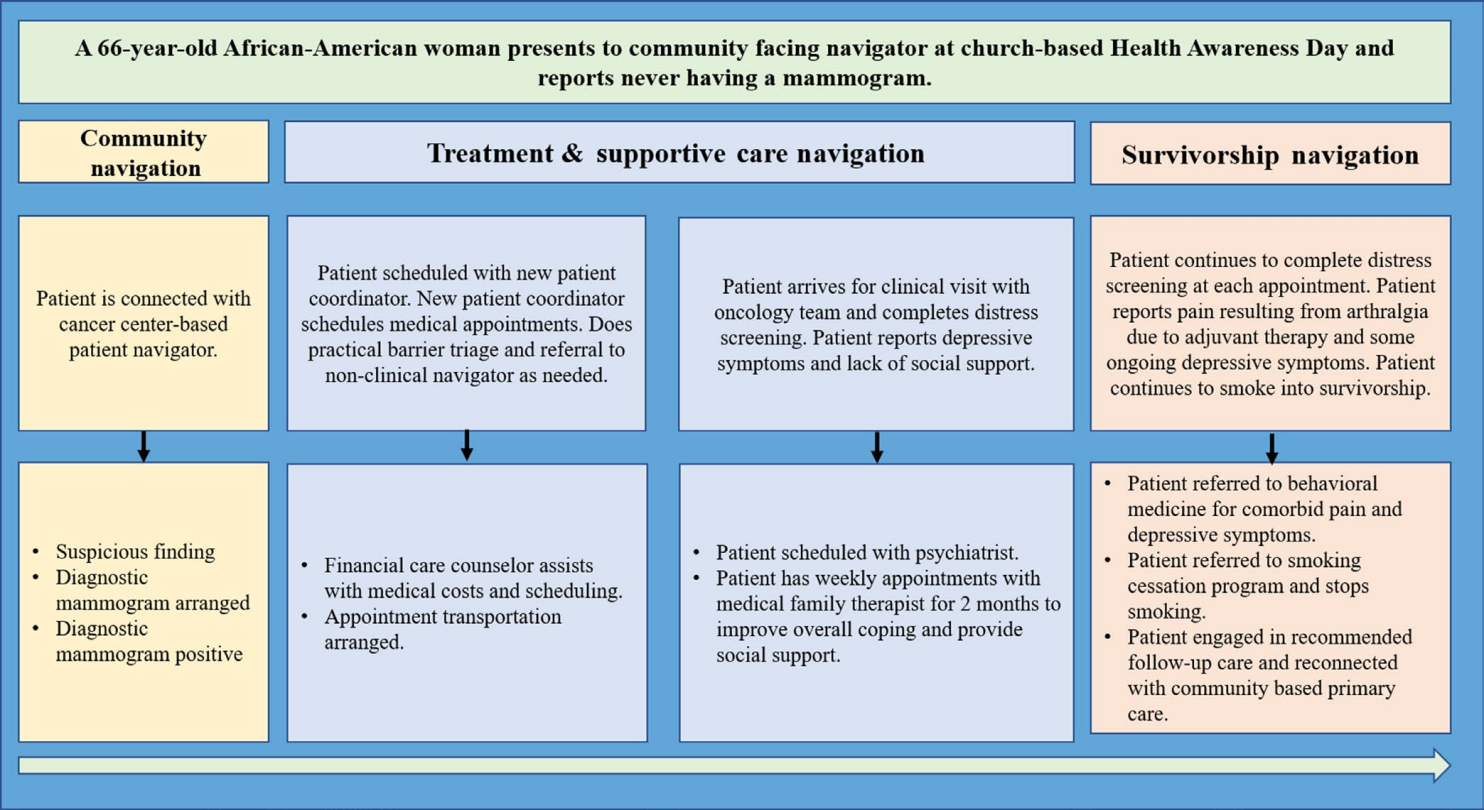
An example of a patient's experience with the longitudinal patient navigation matrix

Distress screening assessments to identify areas of concern and barriers to care are conducted at every provider appointment. This was implemented several years ago as a “sixth vital sign”^27^ and adopted as part of usual care by providers and patients. Such a focus on distress management is now seen in accreditation standards for cancer programs from the American College of Surgeon's Commission on Cancer. Distress screening is conducted for each patient, in the waiting rooms, at each provider visit, creating a longitudinal record of barriers and evolving special needs for every patient (Figure 1). Based on this information, patient needs and barriers undergo a “navigation triage” through which they received navigation services to address practical concerns and/or navigated by either direct connection or referral, to the many financial, psychosocial and supportive care services offered through the Supportive Care and Survivorship Center (Figure 1). Written information on available resources is also provided to the patient with a general number to call if they have questions. In Figure 2 we present a practical and illustrative case example of how a patient moves through the navigation process, from community‐based education on screening to survivorship. Navigators serve as the connective layer to ensure identification of barriers, connection with resources, and longitudinal follow‐up with patients, as appropriate. They connect‐the‐dots as they navigate patients and families to and between services that address logistical, practical, and psychosocial barriers ranging from transportation to financial concerns, to psychological distress, to symptom management.

**Figure 3:**
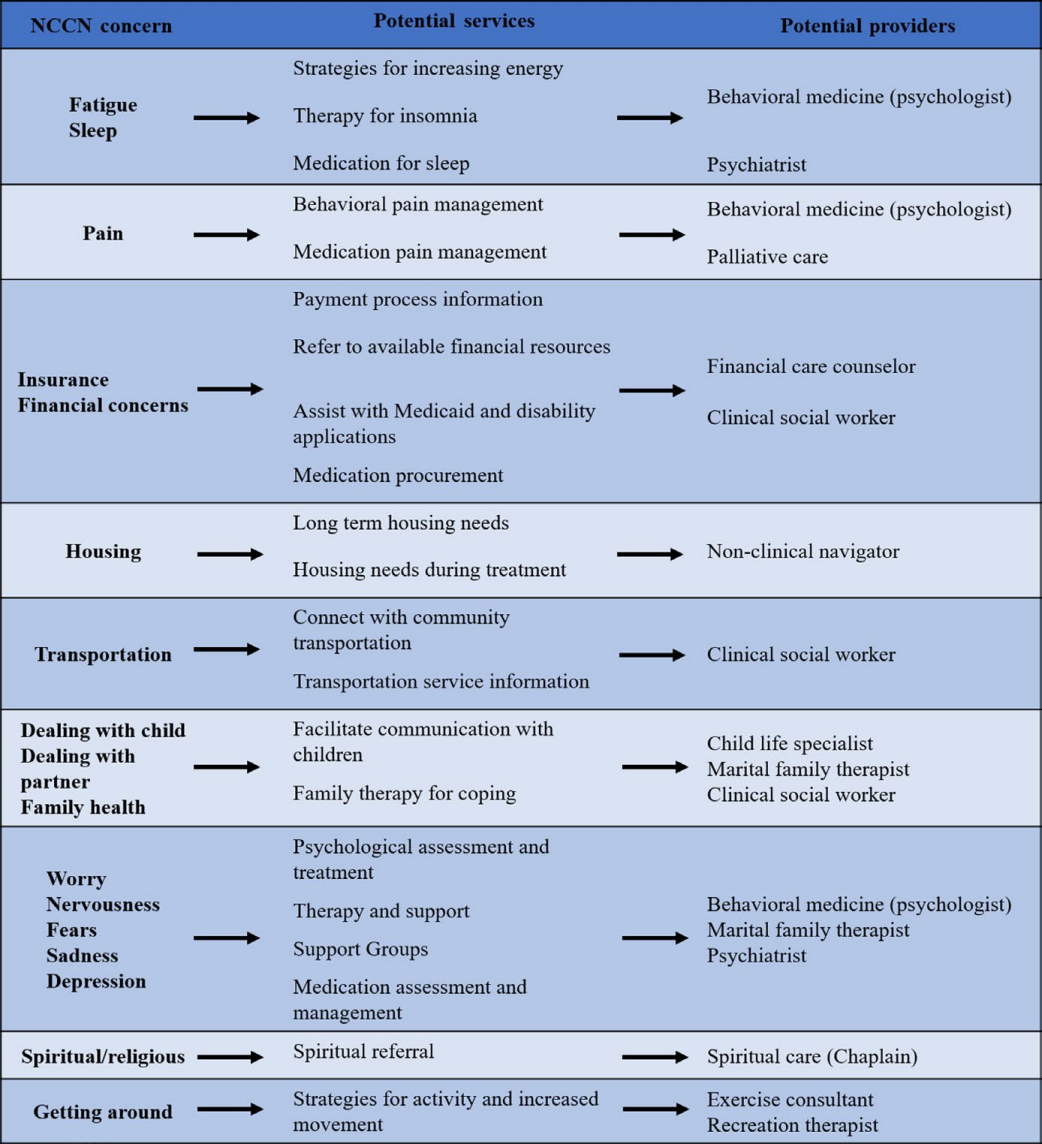
Decision tree for providers for sample of NCCN distress screening concerns

The National Comprehensive Cancer Network's (NCCN) Distress Thermometer and Problem List screening tool, a standardized self‐report measure, is used to identify patient stressors, needs, and barriers. The NCCN Distress Thermometer screens for distress in cancer patients across multiple domains.^28^ First, a single‐item asks patients to rate their distress on an 11‐point visual analog scale ranging from “0” (no distress at all) to “10” (extreme distress) within the past week.^28^, ^29^ Then, the patient completes the Problem List which lists 38 issues, grouped into five categories (ie, practical, family, emotional, spiritual/religious, and physical concerns), to discern the stressors and barriers experienced within the past week.^29^ The information gathered by this instrument is entered in the patient's EMR and navigators and nurses are trained to review the results of the distress screening to further assess needs and refer to services. Oncology nurses and nurse navigators use their clinical judgment to guide practice decisions. If patients rate their distress as a “4” or greater on the visual analog scale, this is considered an evaluation trigger, and the results from the supplemental problem list are used to navigate patients and/or family members to services and resources through the Navigation Model Matrix (Figure 1). There are several check points built into the system to ensure that patients are connected with the appropriate resources; for example, each month a patient navigator receives a list of patients that have reported that transportation is a problem and a follow‐up phone call is made by the navigator to ensure that transportation has not become a barrier to treatment. To guide these connections, and to operationalize the use of the NCCN Distress Thermometer, we developed a decision tree that points to first‐line referral resources for prevalently reported patient NCCN concerns (Figure 3), train all members of the care team on available resources and how to make referrals, and include a metric on our hospital balanced scorecard showing compliance with completing and documenting the distress screening.

As described above, we approach distress screening as a “sixth vital sign”^27^ and it is viewed as part of usual care by both providers and patients. In fiscal year 2018, our compliance for completed distress screening per policy at all locations was over 95%. In fiscal year 2018, 170 291 NCCN distress screens were completed with overall 55% indicating distress and 25% reporting significant distress (ie, ≥4). Among patients who reported significant distress, Table 2 displays the number of concerns reported in each overall domain (ie, practical, family, emotional, spiritual, and physical) and examples of how care is implemented as a result. Among patients who reported any distress on the distress thermometer, the top 5 individual areas of concern were fatigue (58%), pain (48%), worry (37%), nervousness (31%), and sleep (30%). As our compliance with distress screening guidelines has succeeded, our efforts have now shifted to tracking and providing metrics on actions taken (eg, referral placed, resources provided) on distress screening ratings of “4” and above, to ensure we are adequately addressing distress reported by patients. This has been a clinical priority of our team and our goal with tracking is to identify areas where improvement and increased efficiency is needed.

**Table 2:** There were 170 291 NCCN Distress screens completed in FY2018 with 25% reporting significant distress (ie, ≥4). This table displays each distress domain, the number of times concerns in each domain were endorsed among patients with significant distress, and examples of how services were implemented in response to distress

NCCN distress domains	Number of times distress in domain reported	Examples of team member referrals and services implemented
Physical	167 486	Psychologist or research study for behavioral symptom management
		Occupational therapist to assist with activities of daily living
		Sexual health therapist
		Alert oncologist to symptoms
Emotional	73 302	Medical Family Therapist
		Psychologist or psychiatrist
		Support groups
		Teen and Young Adult oncology services
Practical	20 767	Financial care counselor
		Patient Navigator
Family	10 499	Child Life Specialist
		Medical Family Therapist
Spiritual	8970	Chaplain

This system is continually being refined and updated with feedback from patients, family members, and cancer care team members, as well as through utilizing data collected through the tool to expand, refine, and/or target programs. The system also triggers appropriate research programs or trials. For example, we are currently developing a behavioral mobile pain application that can be electronically pushed to the patient based on a pain score of moderate intensity. With such a comprehensive, yet nimble, system, and with the frequency of administration, we are able to identify new stressors and barriers in real time to address accordingly, track and address changes over time, as well as identify eliminated barriers and reduce services, as appropriate.

## CONFLICTS OF INTEREST

The authors SRP and CC have received funding from AstraZeneca for “Digital Innovation in Supportive Care.”

## AUTHOR CONTRIBUTIONS

Cheyenne M. Corbett and Steven R. Patierno were involved in program development, conception and design, literature review, manuscript writing, and final approval. Tamara J. Somers was involved in manuscript writing and final approval. Christine M. Nuñez and Catherine M. Majestic were involved in literature review, manuscript writing, and final approval. Rebecca A. Shelby was involved in conception and design and final approval. Valarie C. Worthy and Nadine J. Barrett were involved in program development, manuscript writing and final approval. 

## Data Availability

This article does not contain data analyses, thus is exempt.
